# *catena*-Poly[[(2,2′-bipyrimidine-κ^2^ *N* ^1^,*N* ^1′^)diperchloratocopper(II)]-μ-4,4′-bipyridine-κ^2^ *N*:*N*′]

**DOI:** 10.1107/S1600536808021296

**Published:** 2008-07-16

**Authors:** Wei Xu, Jian-Li Lin, Hong-Zhen Xie

**Affiliations:** a State Key Laboratory Base of Novel Functional Materials & Prepation Science, Faculty of Materials Science and Chemical Engineering, Ningbo University, Ningbo 315211, People’s Republic of China

## Abstract

The central CuN_4_O_2_ motif of the title compound, [Cu(ClO_4_)_2_(C_8_H_6_N_4_)(C_10_H_8_N_2_)]_*n*_, exhibits a Jahn–Teller-distorted octa­hedral geometry around the metal centre, showing a considerably long Cu—O bond distance of 2.634 (4) Å towards the second perchlorate group occupying the sixth coordination site, giving a (4+1+1)-type coordination mode. The 4,4′-bipyridine (bipy) ligands are highly twisted with respect to each other, the dihedral angle between the two pyridyl ring planes being 38.9 (2)°. The bipy ligands act as bridging ligands between [Cu(ClO_4_)_2_(2,2′-bpym)] (2,2′-bpym is 2,2′-bipyrimidine) units, generating an infinite one-dimensional zigzag chain along [010]. Intra- and intermolecular C—H⋯O hydrogen bonds are present in the crystal structure.

## Related literature

For related literature, see: Biradha & Fujita (2000 ▶); Eddaoudi *et al.* (2001 ▶); Hathaway (1973 ▶); Kaye & Long (2008 ▶); Kitagawa *et al.* (2006 ▶); Subramanian & Zaworotko (1995 ▶).
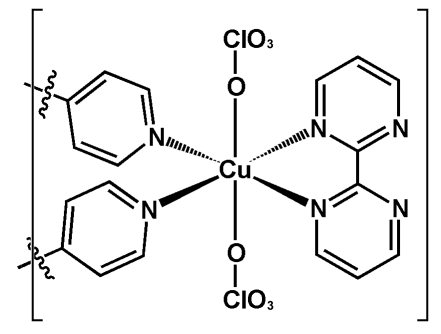


## Experimental

### 

#### Crystal data

[Cu(ClO_4_)_2_(C_8_H_6_N_4_)(C_10_H_8_N_2_)]
*M* *_r_* = 576.75
Monoclinic, 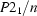

*a* = 11.334 (2) Å
*b* = 14.266 (3) Å
*c* = 13.299 (3) Å
β = 96.55 (3)°
*V* = 2136.3 (8) Å^3^
*Z* = 4
Mo *K*α radiation
μ = 1.33 mm^−1^
*T* = 295 (2) K
0.32 × 0.26 × 0.15 mm

#### Data collection

Bruker P4 diffractometer
Absorption correction: ψ scan (*XSCANS*; Siemens, 1996 ▶) *T*_min_ = 0.664, *T*_max_ = 0.815
4265 measured reflections
3686 independent reflections
2896 reflections with *I* > 2σ(*I*)
*R*_int_ = 0.045
3 standard reflections every 97 reflections intensity decay: none

#### Refinement

*R*[*F* ^2^ > 2σ(*F* ^2^)] = 0.055
*wR*(*F* ^2^) = 0.160
*S* = 1.06
3686 reflections
317 parameters
H-atom parameters constrained
Δρ_max_ = 0.93 e Å^−3^
Δρ_min_ = −0.61 e Å^−3^

### 

Data collection: *XSCANS* (Siemens, 1996 ▶); cell refinement: *XSCANS*; data reduction: *XSCANS*; program(s) used to solve structure: *SHELXS97* (Sheldrick, 2008 ▶); program(s) used to refine structure: *SHELXL97* (Sheldrick, 2008 ▶); molecular graphics: *SHELXL97*; software used to prepare material for publication: *SHELXL97*.

## Supplementary Material

Crystal structure: contains datablocks global, I. DOI: 10.1107/S1600536808021296/im2074sup1.cif

Structure factors: contains datablocks I. DOI: 10.1107/S1600536808021296/im2074Isup2.hkl

Additional supplementary materials:  crystallographic information; 3D view; checkCIF report

## Figures and Tables

**Table 1:** Hydrogen-bond geometry (Å, °)

*D*—H⋯*A*	*D*—H	H⋯*A*	*D*⋯*A*	*D*—H⋯*A*
C1—H1⋯O6^i^	0.93	2.59	3.415 (9)	147
C6—H6⋯O2^ii^	0.93	2.58	3.425 (7)	151
C7—H7⋯O3^iii^	0.93	2.58	3.189 (7)	124
C9—H9⋯O3	0.93	2.56	3.480 (7)	171
C9—H9⋯O4	0.93	2.49	3.185 (6)	132
C11—H11⋯O2^iv^	0.93	2.46	3.342 (6)	159
C16—H16⋯O4^v^	0.93	2.57	3.299 (6)	135
C17—H17⋯O8^v^	0.93	2.47	3.082 (6)	124

Symmetry codes: (i) 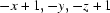; (ii) 
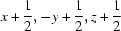
; (iii) 
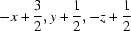
; (iv) 
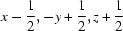
; (v) 
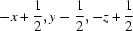
.

**Fig. 1.**
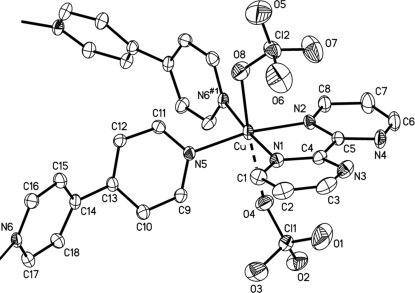
ORTEP view of the title compound. The displacement ellipsoids are drawn at the 40% probability level.

**Fig. 2.**
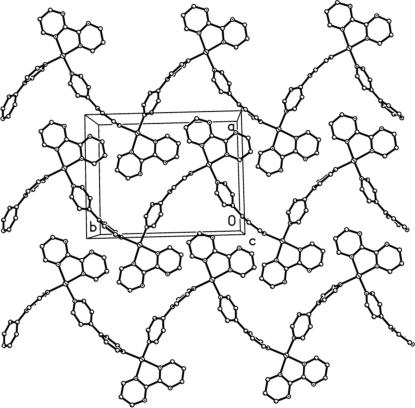
Schematic representation showing the interlocking of 2,2'-bpym rings of two strands resulting in an infinite one-dimensional sheet (perchlorates are omitted for clarity).

## supplementary crystallographic information

### Comment

A great deal of interest in self-assembly of coordination complexes with specific frameworks is expanding rapidly in view of their potentially useful magnetic, catalytic and nonlinear optical properties (Eddaoudi, *et al.*, 2001; Kitagawa, *et al.*, 2006; Kaye & Long, 2008). The ligand 4,4'-bipyridine is an ideal bridging ligand between transition metal atoms to establish coordination networks due to itss two potential binding sites that are arranged in a divergent (*exo*) fashion and its a rigid structure helping to predict network geometries (Subramanian & Zaworotko, 1995; Biradha & Fujita, 2000). 2,2'-bipyrimidine also is a versatile blocking and bridging ligand due to its N_2_ chelating sites on both sides of the ligand. Herein, we report a new complex with one-dimensional zigzag chains, (I), obtained by self-assembly from Cu(ClO_4_)_2_, 4,4'-bpy and 2,2'-bpym in DMF solution. 2,2'-bpym acts as a bidentate ligand with the second chelating site not coodinating the metal atom.

In the title compound, the Cu atom is located in a Jahn-Teller distorted octahedral coordination environment with four N atoms from one 2,2'-bpym ligand (N1, N2) and two 4,4'-bpy ligands [N5, N6^#1^ (#1 = 1/2 - *x*, 1/2 + *y*, 1/2 - *z*)] adopting a planar arrangement (*d*(Cu—N) = 1.998 (4)–2.008 (4) Å). The Cu(II) centre is displaced out of the N_4_ plane by 0.062 (2) Å in the direction of one of perchlorate ligand with d(Cu—O8) = 2.421 (4) Å. The O atom of the second perchlorate group occupies a sixth coordination site at a longer distance of 2.634 (4) Å, completing the overall (4 + 1 + 1) type coordination. O4 is situated slightly off the axial direct of the square pyramid, nevertheless it is close enough to the Cu atom (Hathaway, 1973). The complex can thus be interpreted of consisting of [(2,2'-bpym)Cu(ClO_4_)_2_] units attached to each other *via* 4,4'-bpy to give a zigzag one-dimensional chain along [010] with the chelating 2,2'-bpym ligands extending outwards. The pyrimidine rings of the 2,2'-bpym ligand are twisted relative to each other at 8.7 (1)°, while the dihedral angel of the pyridine rings of the 4,4'-bpy ligand is 38.9 (2)°. Another interesting feature of the structure is that the backbone of the 2,2'-bpym ligand extends sideways from either face of the 4,4'-bpy ribbon and intimately interlocks the interchain region that separates adjacent 4,4'-bpy ribbons (Fig. 2). The perchlorate groups exhibit weak intramolecular hydrogen bonds between the O atoms and the C atoms of the 4,4'-bpy ligands with d(C···O) = 3.082 (6)–3.480 (7) Å and <(C—H···O) = 124–171° (Table 1.). In addition, intermolecular C—H···O hydrogen bonds between the C atoms of the 2,2'-bpym and 4,4'-bpy ligands and the O atoms of the perchlorate groups (*d*(C···O) = 3.189 (7)–3.425 (7) Å and <(C—H···O) = 124–159°) are observed that are responsible for the three-dimensional supramolecular assembly.

### Experimental

Addition of 0.372 g (1.0 mmol) Cu(ClO_4_)_2_, 0.158 g (1.0 mmol) 4,4'-bipyridine and 0.158 g (1.0 mmol) 2,2'-bipyrimidine to a stirred DMF solution (30 ml) yielde a purple precipitate, which was refluxed for 2 h at 403 K followed by filtration after cooling. The resulting light-green filtrate was maintained at room temperature, slow evaporation afforded a small amount of purple block crystals two weeks later.

### Refinement

All H atoms were positioned geometrically and refined as riding atoms, with C—H distances at 0.93 Å and *U*_iso_(H) = 1.2 *U*_eq_(C).

### Crystal data

**Table:**

[Cu(ClO_4_)_2_(C_8_H_6_N_4_)(C_10_H_8_N_2_)]	*F*_000_ = 1164
*M**_r_* = 576.75	*D*_x_ = 1.793 Mg m^−^^3^
Monoclinic, *P*2_1_/*n*	Mo *K*α radiation λ = 0.71073 Å
Hall symbol: -P 2yn	Cell parameters from 25 reflections
*a* = 11.334 (2) Å	θ = 5.0–12.5º
*b* = 14.266 (3) Å	µ = 1.34 mm^−^^1^
*c* = 13.299 (3) Å	*T* = 295 (2) K
β = 96.55 (3)º	Block, purple
*V* = 2136.3 (8) Å^3^	0.32 × 0.26 × 0.15 mm
*Z* = 4	

### Data collection

**Table:**

Bruker P4 diffractometer	*R*_int_ = 0.045
Radiation source: fine-focus sealed tube	θ_max_ = 25.0º
Monochromator: graphite	θ_min_ = 2.1º
*T* = 295(2) K	*h* = −13→1
θ/2θ scans	*k* = −1→16
Absorption correction: multi-scan(XSCANS; Siemens, 1996)	*l* = −15→15
*T*_min_ = 0.664, *T*_max_ = 0.815	3 standard reflections
4265 measured reflections	every 97 reflections
3686 independent reflections	intensity decay: none
2896 reflections with *I* > 2σ(*I*)	

### Refinement

**Table:**

Refinement on *F*^2^	Secondary atom site location: difference Fourier map
Least-squares matrix: full	Hydrogen site location: inferred from neighbouring sites
*R*[*F*^2^ > 2σ(*F*^2^)] = 0.055	H-atom parameters constrained
*wR*(*F*^2^) = 0.160	*w* = 1/[σ^2^(*F*_o_^2^) + (0.0889*P*)^2^ + 4.3786*P*] where *P* = (*F*_o_^2^ + 2*F*_c_^2^)/3
*S* = 1.06	(Δ/σ)_max_ = 0.001
3686 reflections	Δρ_max_ = 0.93 e Å^−^^3^
317 parameters	Δρ_min_ = −0.61 e Å^−^^3^
Primary atom site location: structure-invariant direct methods	Extinction correction: none

### Special details

**Table:**

Geometry. All e.s.d.'s (except the e.s.d. in the dihedral angle between two l.s. planes) are estimated using the full covariance matrix. The cell e.s.d.'s are taken into account individually in the estimation of e.s.d.'s in distances, angles and torsion angles; correlations between e.s.d.'s in cell parameters are only used when they are defined by crystal symmetry. An approximate (isotropic) treatment of cell e.s.d.'s is used for estimating e.s.d.'s involving l.s. planes.
Refinement. Refinement of *F*^2^ against ALL reflections. The weighted *R*-factor *wR* and goodness of fit *S* are based on *F*^2^, conventional *R*-factors *R* are based on *F*, with *F* set to zero for negative *F*^2^. The threshold expression of *F*^2^ > σ(*F*^2^) is used only for calculating *R*-factors(gt) *etc*. and is not relevant to the choice of reflections for refinement. *R*-factors based on *F*^2^ are statistically about twice as large as those based on *F*, and *R*- factors based on ALL data will be even larger.

### Fractional atomic coordinates and isotropic or equivalent isotropic displacement parameters (Å^2^)

**Table:**

	*x*	*y*	*z*	*U*_iso_*/*U*_eq_	
Cu	0.59615 (4)	0.21166 (4)	0.34471 (4)	0.0296 (2)	
Cl1	0.73943 (10)	0.14413 (9)	0.10813 (9)	0.0398 (3)	
Cl2	0.62782 (13)	0.18070 (11)	0.60732 (10)	0.0534 (4)	
N1	0.6774 (3)	0.0876 (3)	0.3709 (3)	0.0331 (8)	
N2	0.7638 (3)	0.2575 (3)	0.3759 (3)	0.0299 (8)	
N3	0.8653 (4)	0.0233 (3)	0.4312 (3)	0.0459 (11)	
N4	0.9612 (3)	0.2015 (3)	0.4100 (4)	0.0460 (11)	
N5	0.4387 (3)	0.1567 (3)	0.2901 (3)	0.0309 (8)	
N6	−0.0235 (3)	−0.1609 (3)	0.1722 (3)	0.0307 (8)	
C1	0.6283 (5)	0.0024 (3)	0.3741 (4)	0.0429 (12)	
H1	0.5471	−0.0046	0.3557	0.051*	
C2	0.6961 (6)	−0.0750 (4)	0.4043 (5)	0.0548 (15)	
H2	0.6629	−0.1346	0.4050	0.066*	
C3	0.8144 (5)	−0.0602 (4)	0.4332 (4)	0.0518 (14)	
H3	0.8614	−0.1113	0.4553	0.062*	
C4	0.7946 (4)	0.0938 (3)	0.3996 (3)	0.0328 (10)	
C5	0.8442 (4)	0.1889 (3)	0.3958 (3)	0.0335 (10)	
C6	0.9985 (4)	0.2895 (4)	0.4055 (5)	0.0528 (15)	
H6	1.0798	0.3009	0.4147	0.063*	
C7	0.9233 (5)	0.3644 (4)	0.3880 (5)	0.0562 (15)	
H7	0.9516	0.4255	0.3858	0.067*	
C8	0.8037 (4)	0.3446 (4)	0.3737 (4)	0.0399 (11)	
H8	0.7496	0.3935	0.3623	0.048*	
C9	0.4280 (4)	0.1042 (4)	0.2051 (4)	0.0386 (11)	
H9	0.4910	0.1029	0.1661	0.046*	
C10	0.3281 (4)	0.0526 (3)	0.1737 (4)	0.0378 (11)	
H10	0.3243	0.0166	0.1151	0.045*	
C11	0.3444 (4)	0.1639 (3)	0.3396 (4)	0.0336 (10)	
H11	0.3486	0.2039	0.3951	0.040*	
C12	0.2399 (4)	0.1153 (3)	0.3132 (4)	0.0326 (10)	
H12	0.1756	0.1228	0.3501	0.039*	
C13	0.2324 (4)	0.0551 (3)	0.2310 (3)	0.0295 (9)	
C14	0.1326 (4)	−0.0117 (3)	0.2077 (4)	0.0316 (10)	
C15	0.0802 (4)	−0.0526 (4)	0.2857 (4)	0.0383 (11)	
H15	0.0956	−0.0296	0.3514	0.046*	
C16	0.0050 (4)	−0.1276 (3)	0.2654 (4)	0.0378 (11)	
H16	−0.0273	−0.1562	0.3188	0.045*	
C17	0.0193 (4)	−0.1170 (3)	0.0946 (4)	0.0348 (10)	
H17	−0.0041	−0.1368	0.0287	0.042*	
C18	0.0976 (4)	−0.0428 (3)	0.1113 (3)	0.0335 (10)	
H18	0.1269	−0.0137	0.0567	0.040*	
O1	0.8332 (5)	0.1061 (6)	0.1735 (4)	0.114 (2)	
O2	0.7807 (4)	0.1965 (3)	0.0279 (3)	0.0648 (12)	
O3	0.6646 (5)	0.0702 (3)	0.0637 (4)	0.0770 (14)	
O4	0.6684 (3)	0.2050 (3)	0.1635 (3)	0.0522 (10)	
O5	0.5763 (7)	0.1934 (7)	0.6933 (5)	0.140 (3)	
O6	0.6507 (6)	0.0798 (4)	0.6044 (5)	0.111 (2)	
O7	0.7399 (5)	0.2209 (5)	0.6042 (5)	0.100 (2)	
O8	0.5503 (4)	0.1986 (3)	0.5175 (3)	0.0596 (11)	

### Atomic displacement parameters (Å^2^)

**Table:**

	*U*^11^	*U*^22^	*U*^33^	*U*^12^	*U*^13^	*U*^23^
Cu	0.0195 (3)	0.0221 (3)	0.0458 (4)	0.00049 (19)	−0.0030 (2)	0.0002 (2)
Cl1	0.0354 (6)	0.0419 (7)	0.0432 (7)	0.0003 (5)	0.0089 (5)	0.0013 (5)
Cl2	0.0532 (8)	0.0679 (9)	0.0384 (7)	−0.0110 (7)	0.0023 (6)	0.0033 (6)
N1	0.034 (2)	0.0248 (19)	0.040 (2)	0.0037 (15)	0.0016 (16)	0.0017 (16)
N2	0.0261 (18)	0.0251 (19)	0.036 (2)	0.0020 (15)	−0.0055 (15)	0.0004 (15)
N3	0.044 (2)	0.043 (2)	0.051 (3)	0.020 (2)	0.007 (2)	0.011 (2)
N4	0.0232 (19)	0.051 (3)	0.061 (3)	0.0069 (18)	−0.0050 (18)	0.000 (2)
N5	0.0236 (17)	0.0280 (19)	0.041 (2)	−0.0027 (15)	0.0019 (15)	−0.0057 (16)
N6	0.0202 (16)	0.0299 (19)	0.041 (2)	−0.0021 (14)	−0.0016 (15)	−0.0023 (16)
C1	0.048 (3)	0.029 (2)	0.051 (3)	−0.003 (2)	0.006 (2)	0.001 (2)
C2	0.077 (4)	0.030 (3)	0.059 (4)	0.007 (3)	0.019 (3)	0.006 (2)
C3	0.069 (4)	0.035 (3)	0.055 (3)	0.023 (3)	0.022 (3)	0.012 (2)
C4	0.035 (2)	0.033 (2)	0.031 (2)	0.0108 (19)	0.0043 (18)	0.0046 (18)
C5	0.028 (2)	0.038 (3)	0.033 (2)	0.0066 (19)	−0.0029 (18)	−0.0033 (19)
C6	0.024 (2)	0.058 (4)	0.073 (4)	−0.005 (2)	−0.003 (2)	−0.008 (3)
C7	0.035 (3)	0.043 (3)	0.088 (5)	−0.008 (2)	−0.004 (3)	−0.004 (3)
C8	0.028 (2)	0.037 (3)	0.053 (3)	−0.001 (2)	−0.002 (2)	−0.003 (2)
C9	0.027 (2)	0.045 (3)	0.046 (3)	−0.008 (2)	0.011 (2)	−0.011 (2)
C10	0.030 (2)	0.039 (3)	0.045 (3)	−0.007 (2)	0.005 (2)	−0.007 (2)
C11	0.031 (2)	0.026 (2)	0.043 (3)	−0.0042 (18)	0.0029 (19)	−0.0080 (19)
C12	0.027 (2)	0.032 (2)	0.039 (2)	−0.0031 (18)	0.0076 (18)	−0.0071 (19)
C13	0.024 (2)	0.027 (2)	0.037 (2)	−0.0026 (17)	0.0000 (17)	−0.0003 (18)
C14	0.0207 (19)	0.029 (2)	0.045 (3)	−0.0007 (17)	0.0016 (18)	−0.0006 (19)
C15	0.031 (2)	0.042 (3)	0.042 (3)	−0.010 (2)	0.0049 (19)	−0.005 (2)
C16	0.033 (2)	0.041 (3)	0.040 (3)	−0.011 (2)	0.011 (2)	−0.002 (2)
C17	0.032 (2)	0.032 (2)	0.038 (3)	−0.0027 (19)	−0.0050 (19)	0.000 (2)
C18	0.034 (2)	0.033 (2)	0.032 (2)	−0.0057 (19)	−0.0001 (18)	0.0025 (19)
O1	0.077 (3)	0.183 (7)	0.078 (4)	0.063 (4)	−0.006 (3)	0.023 (4)
O2	0.071 (3)	0.064 (3)	0.065 (3)	−0.014 (2)	0.036 (2)	0.002 (2)
O3	0.089 (3)	0.047 (2)	0.099 (4)	−0.023 (2)	0.028 (3)	−0.021 (2)
O4	0.052 (2)	0.053 (2)	0.055 (2)	−0.0009 (18)	0.0210 (18)	−0.0097 (18)
O5	0.116 (5)	0.254 (10)	0.054 (3)	0.016 (6)	0.026 (3)	−0.011 (5)
O6	0.126 (5)	0.068 (4)	0.131 (5)	0.011 (3)	−0.023 (4)	0.034 (4)
O7	0.083 (4)	0.130 (5)	0.083 (4)	−0.041 (3)	−0.004 (3)	0.015 (3)
O8	0.056 (2)	0.078 (3)	0.044 (2)	0.021 (2)	0.0044 (18)	0.012 (2)

### Geometric parameters (Å, °)

**Table:**

Cu—N6^i^	1.998 (4)	C1—H1	0.9300
Cu—N1	2.007 (4)	C2—C3	1.369 (9)
Cu—N2	2.008 (4)	C2—H2	0.9300
Cu—N5	2.008 (4)	C3—H3	0.9300
Cu—O8	2.421 (4)	C4—C5	1.471 (7)
Cu—O4	2.634 (4)	C6—C7	1.370 (8)
Cl1—O1	1.403 (5)	C6—H6	0.9300
Cl1—O2	1.424 (4)	C7—C8	1.377 (7)
Cl1—O3	1.438 (4)	C7—H7	0.9300
Cl1—O4	1.442 (4)	C8—H8	0.9300
Cl2—O5	1.354 (6)	C9—C10	1.376 (6)
Cl2—O7	1.399 (6)	C9—H9	0.9300
Cl2—O8	1.422 (4)	C10—C13	1.395 (6)
Cl2—O6	1.464 (6)	C10—H10	0.9300
N1—C1	1.339 (6)	C11—C12	1.383 (6)
N1—C4	1.343 (6)	C11—H11	0.9300
N2—C8	1.323 (6)	C12—C13	1.385 (6)
N2—C5	1.343 (6)	C12—H12	0.9300
N3—C4	1.325 (6)	C13—C14	1.484 (6)
N3—C3	1.325 (7)	C14—C18	1.372 (6)
N4—C6	1.329 (7)	C14—C15	1.382 (7)
N4—C5	1.331 (6)	C15—C16	1.376 (7)
N5—C11	1.321 (6)	C15—H15	0.9300
N5—C9	1.350 (6)	C16—H16	0.9300
N6—C16	1.332 (6)	C17—C18	1.383 (6)
N6—C17	1.344 (6)	C17—H17	0.9300
N6—Cu^ii^	1.998 (4)	C18—H18	0.9300
C1—C2	1.379 (7)		
			
N6^i^—Cu—N1	175.53 (15)	C2—C3—H3	118.4
N6^i^—Cu—N2	95.45 (14)	N3—C4—N1	125.7 (4)
N1—Cu—N2	81.19 (15)	N3—C4—C5	119.4 (4)
N6^i^—Cu—N5	88.66 (15)	N1—C4—C5	114.9 (4)
N1—Cu—N5	95.15 (15)	N4—C5—N2	124.9 (5)
N2—Cu—N5	169.48 (15)	N4—C5—C4	119.9 (4)
N6^i^—Cu—O8	92.65 (15)	N2—C5—C4	115.2 (4)
N1—Cu—O8	84.89 (15)	N4—C6—C7	123.4 (5)
N2—Cu—O8	97.45 (15)	N4—C6—H6	118.3
N5—Cu—O8	92.00 (15)	C7—C6—H6	118.3
N6^i^—Cu—O4	95.57 (14)	C6—C7—C8	116.6 (5)
N1—Cu—O4	86.74 (14)	C6—C7—H7	121.7
N2—Cu—O4	79.35 (14)	C8—C7—H7	121.7
N5—Cu—O4	90.64 (14)	N2—C8—C7	121.5 (5)
O8—Cu—O4	171.41 (13)	N2—C8—H8	119.2
O1—Cl1—O2	112.1 (4)	C7—C8—H8	119.2
O1—Cl1—O3	109.9 (4)	N5—C9—C10	122.9 (4)
O2—Cl1—O3	107.8 (3)	N5—C9—H9	118.5
O1—Cl1—O4	110.2 (3)	C10—C9—H9	118.5
O2—Cl1—O4	108.5 (3)	C9—C10—C13	119.0 (4)
O3—Cl1—O4	108.3 (3)	C9—C10—H10	120.5
O5—Cl2—O7	116.8 (5)	C13—C10—H10	120.5
O5—Cl2—O8	113.6 (4)	N5—C11—C12	123.5 (4)
O7—Cl2—O8	112.2 (3)	N5—C11—H11	118.2
O5—Cl2—O6	104.4 (5)	C12—C11—H11	118.2
O7—Cl2—O6	103.8 (4)	C11—C12—C13	119.0 (4)
O8—Cl2—O6	104.4 (3)	C11—C12—H12	120.5
C1—N1—C4	116.9 (4)	C13—C12—H12	120.5
C1—N1—Cu	128.4 (3)	C12—C13—C10	117.7 (4)
C4—N1—Cu	114.2 (3)	C12—C13—C14	122.6 (4)
C8—N2—C5	117.6 (4)	C10—C13—C14	119.4 (4)
C8—N2—Cu	128.2 (3)	C18—C14—C15	117.5 (4)
C5—N2—Cu	114.1 (3)	C18—C14—C13	122.3 (4)
C4—N3—C3	116.1 (5)	C15—C14—C13	119.8 (4)
C6—N4—C5	116.0 (4)	C16—C15—C14	119.3 (5)
C11—N5—C9	117.4 (4)	C16—C15—H15	120.3
C11—N5—Cu	121.7 (3)	C14—C15—H15	120.3
C9—N5—Cu	120.7 (3)	N6—C16—C15	122.6 (4)
C16—N6—C17	118.6 (4)	N6—C16—H16	118.7
C16—N6—Cu^ii^	118.8 (3)	C15—C16—H16	118.7
C17—N6—Cu^ii^	121.2 (3)	N6—C17—C18	121.0 (4)
N1—C1—C2	121.1 (5)	N6—C17—H17	119.5
N1—C1—H1	119.5	C18—C17—H17	119.5
C2—C1—H1	119.5	C14—C18—C17	120.6 (4)
C3—C2—C1	117.0 (5)	C14—C18—H18	119.7
C3—C2—H2	121.5	C17—C18—H18	119.7
C1—C2—H2	121.5	Cl1—O4—Cu	137.6 (2)
N3—C3—C2	123.2 (5)	Cl2—O8—Cu	129.2 (2)
N3—C3—H3	118.4		

Symmetry codes: (i) −*x*+1/2, *y*+1/2, −*z*+1/2; (ii) −*x*+1/2, *y*−1/2, −*z*+1/2.

### Hydrogen-bond geometry (Å, °)

**Table:**

*D*—H···*A*	*D*—H	H···*A*	*D*···*A*	*D*—H···*A*
C1—H1···O6^iii^	0.93	2.59	3.415 (9)	147
C6—H6···O2^iv^	0.93	2.58	3.425 (7)	151
C7—H7···O3^v^	0.93	2.58	3.189 (7)	124
C9—H9···O3	0.93	2.56	3.480 (7)	171
C9—H9···O4	0.93	2.49	3.185 (6)	132
C11—H11···O2^vi^	0.93	2.46	3.342 (6)	159
C16—H16···O4^ii^	0.93	2.57	3.299 (6)	135
C17—H17···O8^ii^	0.93	2.47	3.082 (6)	124

Symmetry codes: (iii) −*x*+1, −*y*, −*z*+1; (iv) *x*+1/2, −*y*+1/2, *z*+1/2; (v) −*x*+3/2, *y*+1/2, −*z*+1/2;  (vi) *x*−1/2, −*y*+1/2, *z*+1/2; (ii) −*x*+1/2, *y*−1/2, −*z*+1/2.
